# Identification of miRNA-eQTLs in maize mature leaf by GWAS

**DOI:** 10.1186/s12864-020-07073-0

**Published:** 2020-10-06

**Authors:** Shu-Yun Chen, Mei-Hsiu Su, Karl A. Kremling, Nicholas K. Lepak, M. Cinta Romay, Qi Sun, Peter J. Bradbury, Edward S. Buckler, Hsin-Mei Ku

**Affiliations:** 1 Department of Life Science, National Cheng Kung University, Tainan, 701 Taiwan; 2 Agricultural Biotechnology Research Center, Academia Sinica, Taipei, 115 Taiwan; 3 Department of Plant Breeding and Genetics, Cornell University, Ithaca, 14850 NY USA; 4 United States Department of Agriculture-Agricultural Research Service, Ithaca, NY USA; 5 Institute for Genomic Diversity, Cornell University, Ithaca, NY USA; 6 Advanced Plant Biotechnology Center, National Chung Hsing University, No 145 Xingda Rd, South Dist, Taichung, 402 Taiwan

**Keywords:** miRNA, miR-eQTLs, Hapmap, Maize, GWAS, miRNA target gene regulation

## Abstract

**Background:**

MiRNAs play essential roles in plant development and response to biotic and abiotic stresses through interaction with their target genes. The expression level of miRNAs shows great variations among different plant accessions, developmental stages, and tissues. Little is known about the content within the plant genome contributing to the variations in plants. This study aims to identify miRNA expression-related quantitative trait loci (miR-QTLs) in the maize genome.

**Results:**

The miRNA expression level from next generation sequencing (NGS) small RNA libraries derived from mature leaf samples of the maize panel (200 maize lines) was estimated as phenotypes, and maize Hapmap v3.2.1 was chosen as the genotype for the genome-wide association study (GWAS). A total of four significant miR-eQTLs were identified contributing to miR156k-5p, miR159a-3p, miR390a-5p and miR396e-5p, and all of them are trans-eQTLs. In addition, a strong positive coexpression of miRNA was found among five miRNA families. Investigation of the effects of these miRNAs on the expression levels and target genes provided evidence that miRNAs control the expression of their targets by suppression and enhancement.

**Conclusions:**

These identified significant miR-eQTLs contribute to the diversity of miRNA expression in the maize penal at the developmental stages of mature leaves in maize, and the positive and negative regulation between miRNA and its target genes has also been uncovered.

## Background

MicroRNAs (miRNAs) are a class of small RNA fragments widely distributed in plants and animal genomes that regulate their target genes through translational inhibition, mature messenger RNA cleavage, and RNA-dependent DNA methylation (RdDM) [1]. In plants, pri-miRNA is transcribed by RNA polymerase II from the miRNA gene with a 5′ cap and 3′ polyadenylation modification and subsequently removed by a microprocessor while cutting the end of the stem-loop structure to produce pre-miRNA. Subsequently, Dicer like-1 (DCL-1) and Dicer like-4 (DCL-4) nucleases cut the pre-miRNA into 21–24 nt short double-strand RNAs, which are methylated by protein Hua-Enhancer1 (HEN1) to produce mature miRNA. The mature miRNA is later exported to the cytoplasm by HASTY (HST) protein. In the cytoplasm, only a single strand of the mature miRNA is loaded into the RNA-induced ribonucleoprotein silencing complex (RISC), which includes Argonaute (AGO1), to guide the miRNA by finding its target sites to cleave the corresponding mRNA or to inhibit translation [2]. In plants, miRNAs regulate target genes by being nearly complementary to the target sequences. In contrast, in animals, they regulate target regions by relying on the recognition of 7–8 nucleotide “seed sequences” [3]. The identification of miRNAs and their target genes has been well documented in both plants [4] and animals [5]. As far as the strategy is concerned, methods such as microarray, miRNA silencing and immunoprecipitation (IP)-based approaches and computational prediction after high-throughput sequencing are also applied for miRNA target identification [6].

The target gene number of miRNAs has been reported to be much lower in plants than in animals. Most of the targets are transcription factors, indicating the importance of plant miRNAs in gene regulation [7, 8]. The mechanisms of miRNA regulating different plant developmental stages have been reported, including (1) posttranscriptional regulation, e.g., miR160 and miR166 regulate the plant height of *Gossypium hirsutum* through auxin and ABA response factors [9]. In Arabidopsis, phosphate starvation regulated by miR399 suppressed its target by binding to the 5′ untranslated region (UTR) [10]. An increase in related miRNAs to regulate response genes under biotic and abiotic stresses has also been reported [11]. (2) Posttranslational regulation: miR172 adjusted the flowering time by inhibiting the translation of flowering gene AP2 (APETALA2) in Arabidopsis [12]. Recently, in maize, many miRNAs and their targets have been identified by computational prediction, such as miRNAs involved in maize kernel development [13, 14] and grain filling stages [15]. In addition, the global repression of miRNA expression has been reported in maize hybrid lines, which may be critical for increasing yield [16, 17]. These reports demonstrate the important function of miRNAs in plants [7].

Numerous miRNA studies have been reported in maize due to its importance as a major source of food starch worldwide. Whole genome miRNA expression profiles from roots, seedlings, tassels, ears, and pollen grains have been reported, in which approximately 35% of ancestral sites were found as duplicated homoeologous miRNAs [18]. Additionally, miRNAs responding to various stresses in different tissues in maize have been reported. The miRNA responses to long-term waterlogging in seedlings [19]; low phosphorus-related miRNAs [20], and low nitrate-related miRNAs [21, 22] in maize seedling roots; and the miRNAs associated with the resistance of *Exserohilum turcicum* [23], and diazotrophic bacteria [24]. All these studies have mainly focused on the identification of miRNAs, miRNA target genes, and miRNA functions by computational prediction. However, it remains unclear how genomic loci affect miRNA expression in the maize genome.

Genome-wide association study (GWAS) has been known as an efficient way to search the locations associated with traits of interest, in particular, quantitative traits. It is similar to QTL mapping, which considers the relationship between genotypes of each marker and the variance of corresponding phenotypes. However, GWAS provides higher resolution than QTL mapping because more recombination events have occurred in the breeding history of tested populations [25, 26]. GWAS has been broadly applied to analyze the correlation between different plant species and hundreds of target traits [27], including agro climatic traits in sorghum [28], carbon isotope ratio in soybean [29], and the association of genome location with head smut resistance in cotton [30]. In addition, when target gene expression was input as a phenotype in GWAS processing, the locations associated with the variations of gene expression could be found as expressed QTLs (eQTLs) in the genome [31]. This approach has been applied in a previous study in which maize root tissue genotyped by 1731 SNPs and the gene expression profile from maize microarray as phenotypes, many cis- and trans-regulators have been successfully identified as causes affecting the gene expression patterns in maize root [32]. In addition, in humans, miRNA expression-related quantitative trait loci (miR-eQTLs) in different organs or stimulations have been reported, including human fibroblasts [33], glioblastoma [34], adipose tissue [35], infected dendritic cells [36], and 5239 human whole blood samples [37].

Although the locations and functions of miRNAs in plants have been revealed by numerous studies, to the best of our knowledge, none of them have focused on miR-eQTLs in plants. Therefore, the relationship between eQTLs responsible for variations in miRNA expression levels in a plant population remains unknown. The objective of this study is to reveal this relationship by combining the maize HapMap and miRNA expression profile of a maize panel (200 maize lines) to identify miR-eQTLs. This is the first report on the identification of four miR-eQTLs in maize and the finding that miRNAs regulate their target genes in both negative and positive ways.

## Result

### Identification of the highly expressed miRNAs in the maize panel

**Fig. 1:**
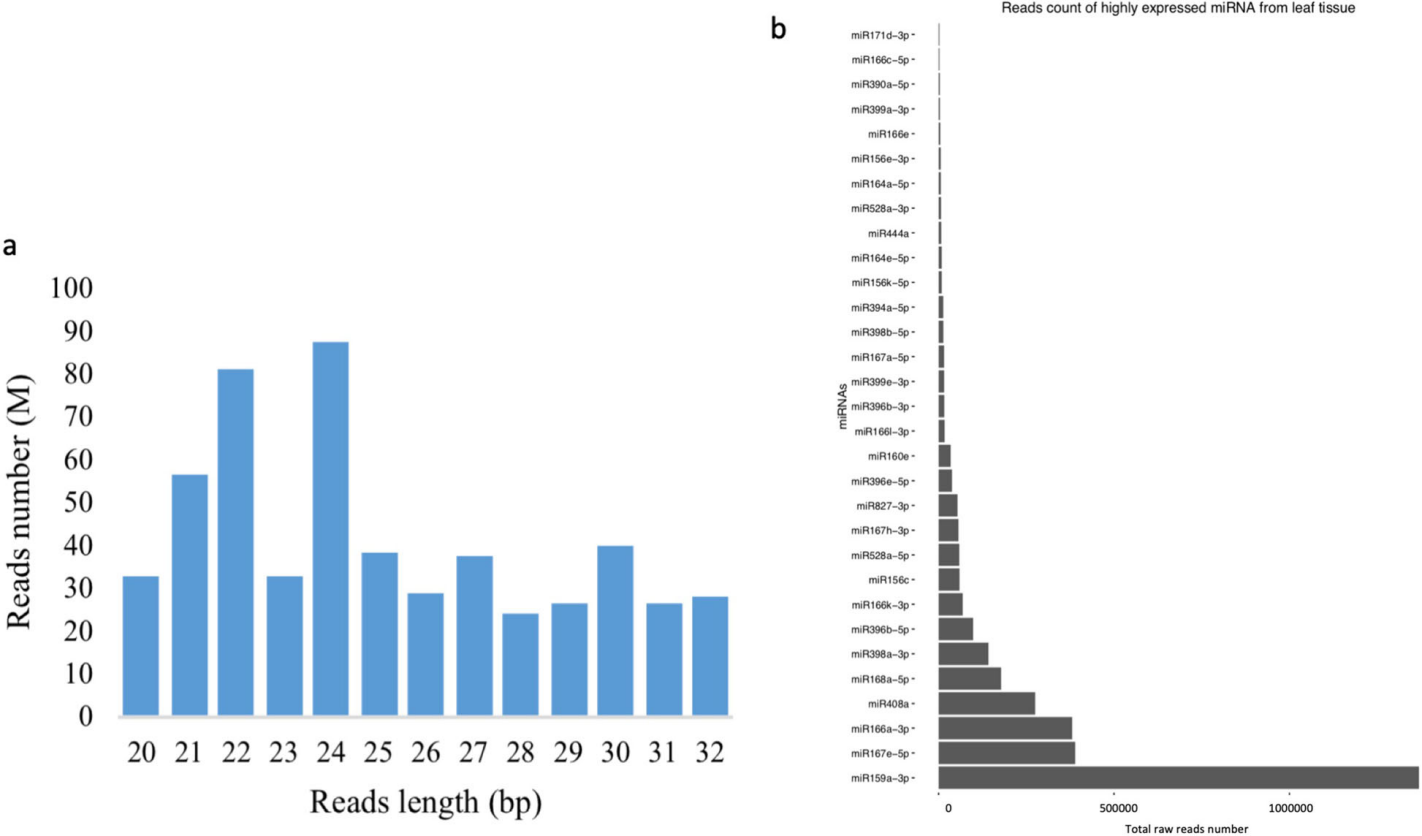
Reads length distribution from 200 maize leaf libraries. a. Reads distribution based on reads length. b. Reads distribution based on different miRNAs

To obtain the miRNA expression data among the 200 maize lines (hereafter called the maize panel), small RNA (sRNA) libraries were developed individually and pooled together for sequencing. Reads with lengths ranging from 20 nt to 32 nt after trimming were kept. The distribution from total libraries showed that 24 nt, 22 nt and 21 nt sRNAs were most abundant (Fig. 1a). This distribution is similar to the previous report by Liu [38], in which the highest peak at read length was 24 nt in an sRNA library from maize developing ear tissue. This indicated that the library developed in this study is suitable for the following analysis. A total of 31 miRNAs belonging to 17 miRNA families were identified and defined as highly expressed miRNAs based on the sum of total read counts greater than 2000 among 200 maize lines of the panel (Fig. 1b). Among highly expressed miRNAs, miR159a-3p showed the highest expression level with 1,369,775 reads, and miR171d-3p had the lowest expression level with 2160 reads.

**Fig. 2:**
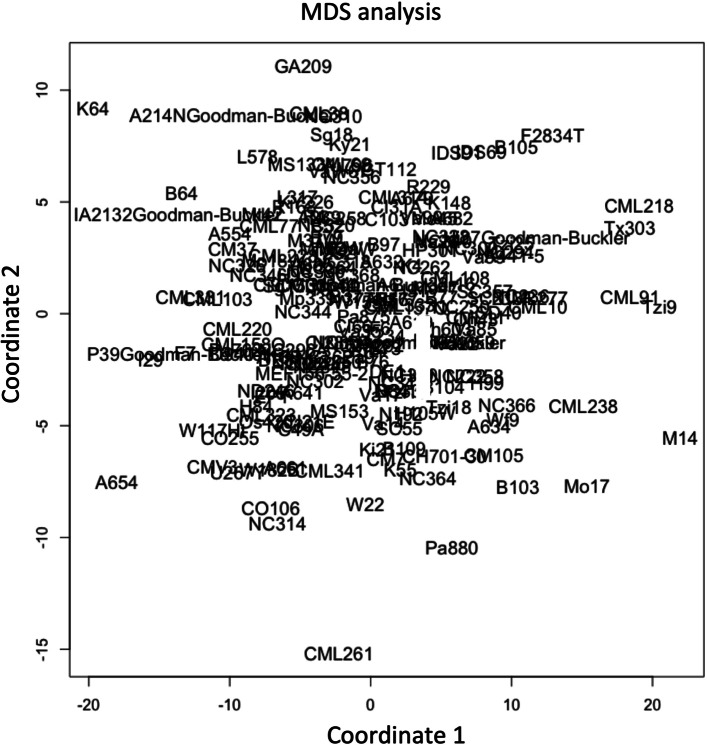
MDS analysis based on 31 highly expressed miRNAs

To understand the diversity of miRNA expression patterns in the maize panel, 31 highly expressed miRNAs were analyzed by multidimensional scaling (MDS) analysis, and no cluster was detected (Fig. 2). Furthermore, the Boxcox transformation was applied for highly expressed miRNAs individually, which transformed the distribution of miRNA expression to a normal distribution for GWAS analysis.

### The coexpression of highly expressed miRNAs in the maize panel

To study the correlation among 31 highly expressed miRNAs, ten combinations including five miRNA families with a correlation higher or equal to 0.7 and a *p*-value lower than 0.001 were identified (Fig. S1). As shown in Table 1, six out of ten combinations belonging to the same miRNA families showed a significant correlation. This included four combinations from the miRNA166 family (miR166a-3p vs miR166e, miR166e vs miR166k-3p, miR166a-3p vs miR166I-3p, miR166k-3p vs miR166I-3p), one combination from the miR167 family (miR167a-5p vs miR167e-5p) and one combination from the miR398 family (miR398a-3p vs miR398b-5p). In addition, four combinations from different miRNA families showed correlations, including miR166k-3p vs miR167h-3p, miR398-3p vs miR408a, miR398a-3p vs miR528a-5p, and miR408a vs miR528a-5p. Among these ten combinations, the highest correlation (0.827) was detected within the miR167 family (miR167a-5p vs miR167e-5p), and the lowest (0.701) was found between the miR398 and miR408 families (miR398a-3p vs miR408a). All ten correlated combinations of highly expressed miRNA loci were not located on the same chromosome (Table 1).

**Table 1:** List of high correlation among 31 highly expressed miRNA (*R*^2^ > 0.7)

Within the same miRNA family					
Variable A	Chr^a^	Variable B	Chr	*R*^2^	*p*-value
miR166a-3p	6	miR166e	4	0.778	< 0.001
miR166e	4	miR166k-3p	5	0.740	< 0.001
miR166a-3p	6	miR166l-3p	3	0.741	< 0.001
miR166k-3p	5	miR166l-3p	3	0.703	< 0.001
miR167a-5p	3	miR167e-5p	7	0.827	< 0.001
miR398a-3p	2	miR398b-5p	7	0.723	< 0.001
**Between different miRNA families**					
miR166k-3p	5	miR167h-3p	6	0.766	< 0.001
miR398a-3p	2	miR408a	8	0.701	< 0.001
miR398a-3p	2	miR528a-5p	1	0.741	< 0.001
miR408a	8	miR528a-5p	1	0.719	< 0.001

^a^ chromosome

### Identification of miR-eQTLs by GWAS analysis

**Fig. 3:**
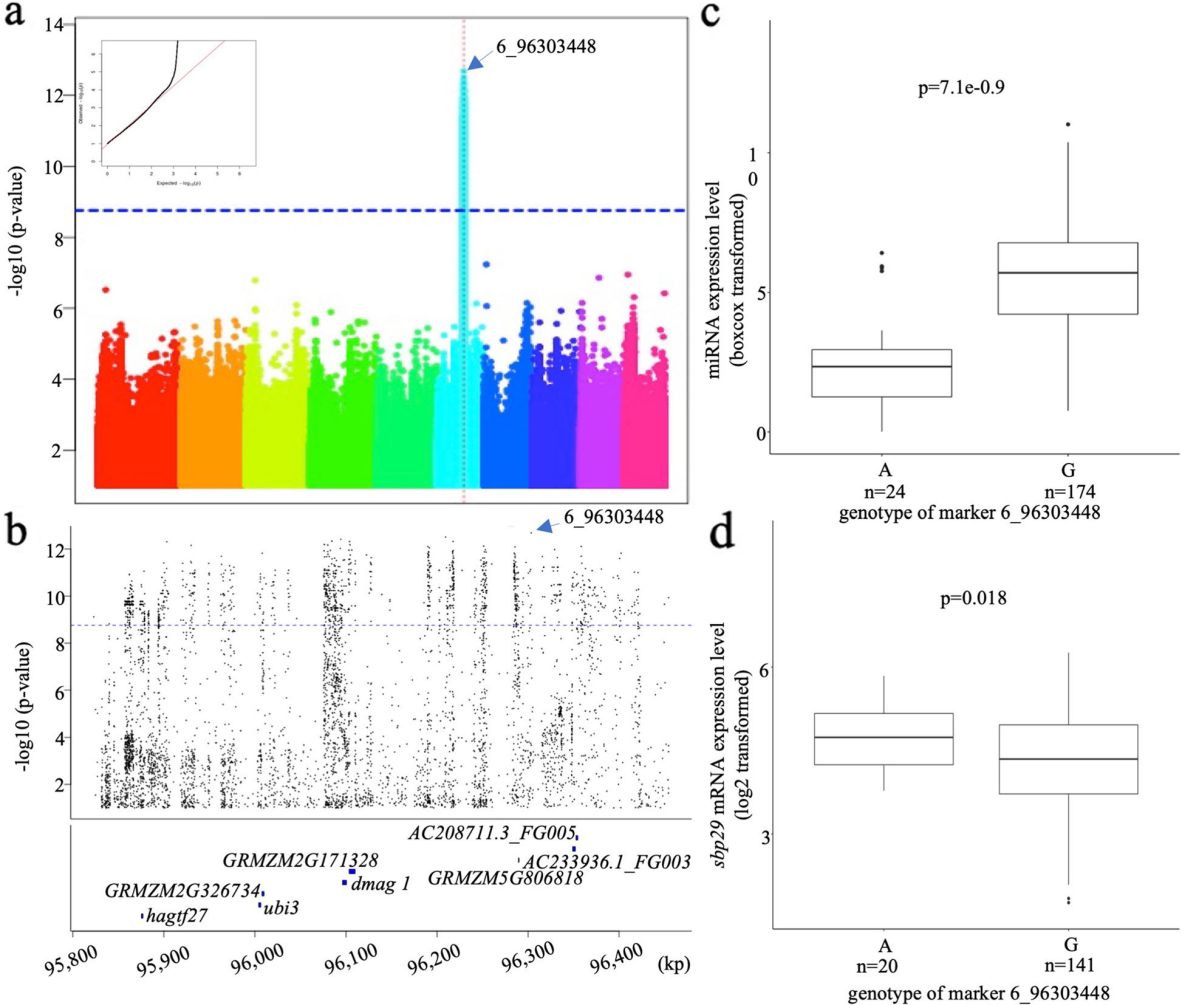
GWAS result of miR156k-5p. a. Manhattan plot of miR156k-5p. b. Genes located within the strong associated region of miR156k-5p. c. The difference of miRNA expression level of miR156k-5p distinguished by most significant SNP type. d. The difference of sbp29 expression level distinguished by most significant SNP type. Red straight dotted line indicates the location of miRNA

**Fig. 4:**
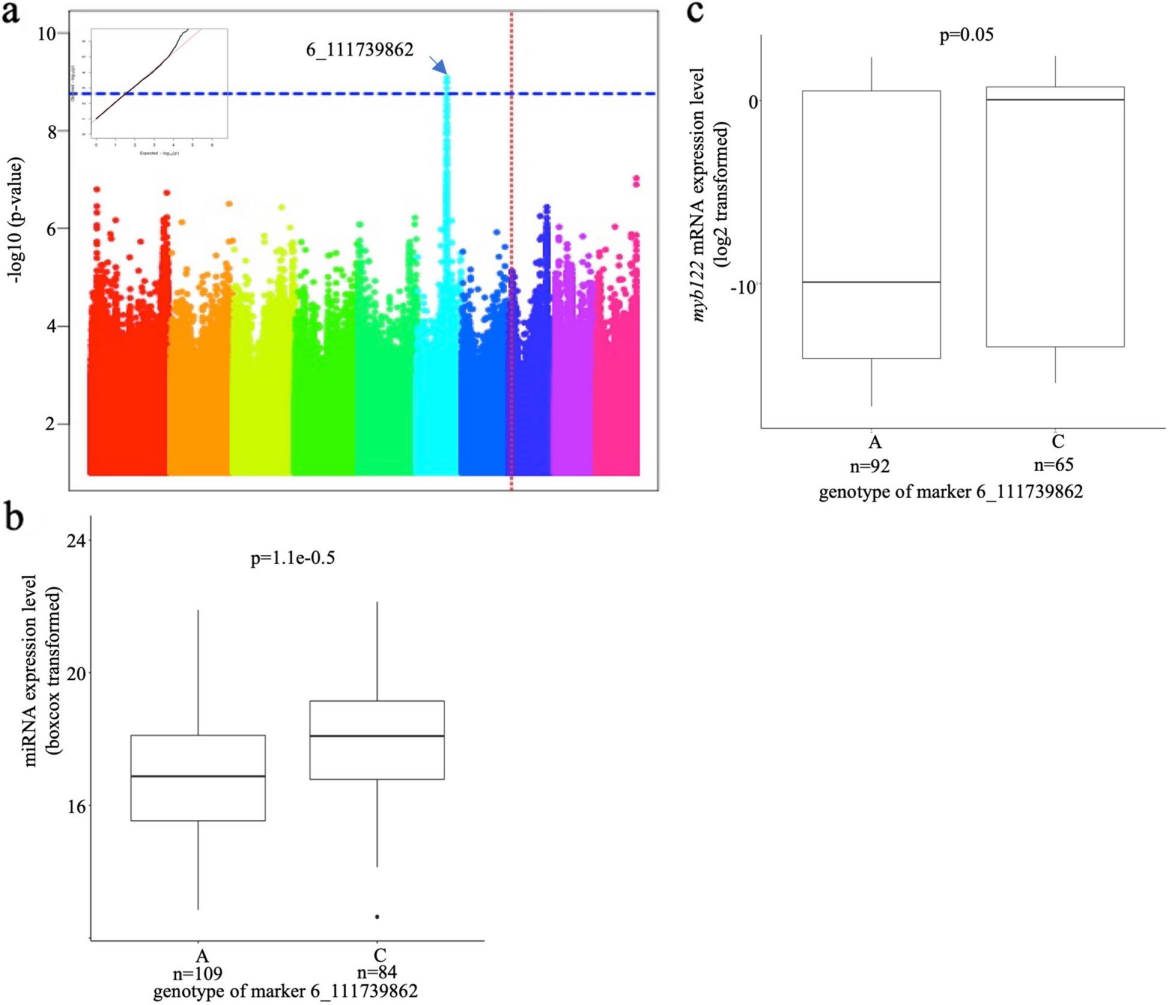
GWAS result of miR159a-3p. a. Manhattan plot of miR159a-3p. b. The difference of miRNA expression level of miR159a-3p distinguished by most significant SNP type. c. The difference of myb122 expression level distinguished by most significant SNP type. Red straight dotted line indicates the location of miRNA

**Fig. 5:**
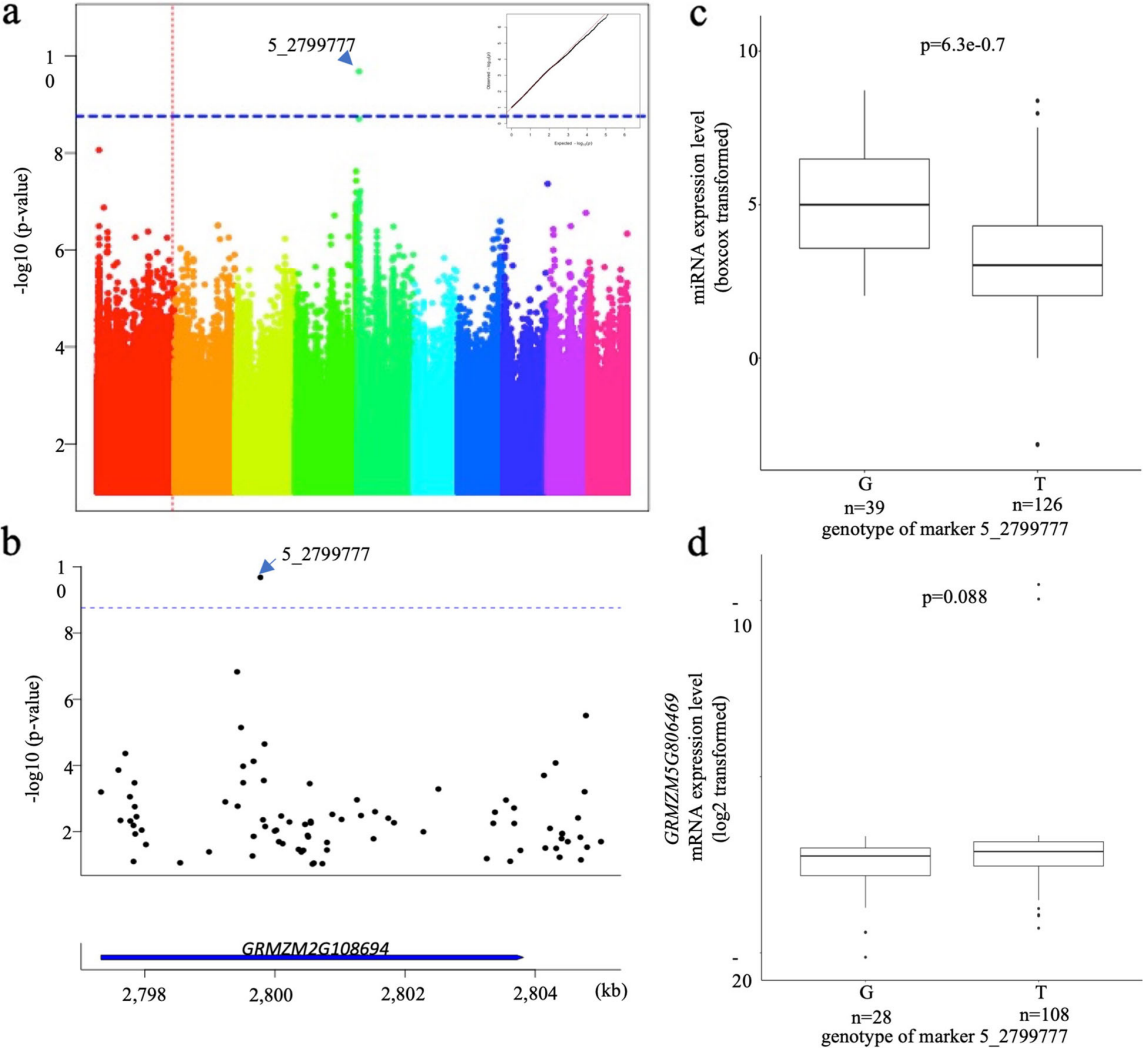
GWAS result of miR390a-5p. a. Manhattan plot of miR390a-5p. b. Genes located within the strong associated region of miR390a-5p. c. The difference of miRNA expression level of miR390a-5p distinguished by most significant SNP type. d. The difference of GRMZM5G806469 expression level distinguished by most significant SNP type. Red straight dotted line indicates the location of miRNA

**Fig. 6:**
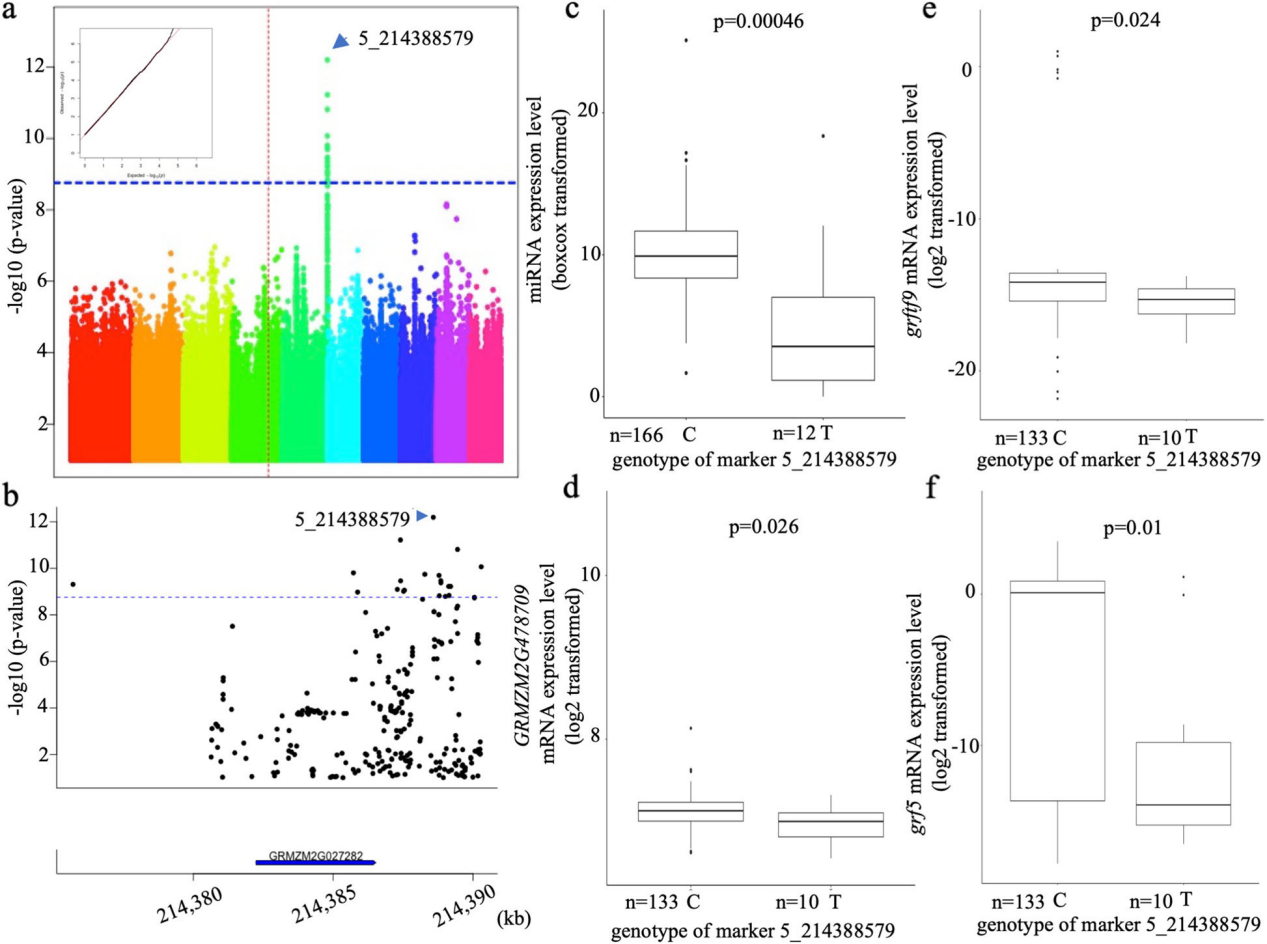
GWAS result of miR396e-5p. a. Manhattan plot of miR396e-5p. b. Genes located within the strong associated region of miR396e-5p. c. The difference of miRNA expression level of miR396e-5p distinguished by most significant SNP type. d. The difference of GRMZM2G478709 expression level distinguished by most significant SNP type. e. The difference of grftf9 expression level distinguished by most significant SNP type. f. The difference of grf5 expression level distinguished by most significant SNP type. Red straight dotted line indicates the location of miRNA

The expression level of the 31 highly expressed miRNAs was input as phenotype data in GWAS analysis based on general linear model (GLM) plus 5 PCA and 2 latent PCA to control the population structure, and the Bonferroni correction level was used as criteria to identify miR-eQTLs. Four miR-eQTLs contributing to miR156k-5p (Fig. 3), miR159a-3p (Fig. 4), miR390a-5p (Fig. 5), and miR396e-5p (Fig. 6) were found individually, which showed a significant association with phenotypes (miRNA expression level) in the panel after GWAS analysis (Table 2). All genes located within miR-eQTLs are summarized in Table 3. First, a highly significant miR-eQTL was found for miR156k-5p at 96303448 bp of chromosome 6 (Fig. 3a, Table 2). Within this genomic region, there were eight genes, including GRMZM2G049730 (GNAT-transcription factor 27, hagtf27), GRMZM2G014119 (ubiquitin3), GRMZM2G326734, GRMZM2G171317 (DNA-3-methyladenine glycosylase1), GRMZM2G171328, GRMZM5G806818, AC233936.1_FG003 and AC208711.3_FG005 (Fig. 3b). Second, another miR-eQTL was detected for mi159a-3p at 111739862 bp on chromosome 6 (Fig. 4a), but no gene was located within this region. Another two miR-eQTLs contributing to miR390a-5p (Fig. 5a) and miR396e-5p (Fig. 6a) were identified, and two genes, GRMZM2G108694 and GRMZM2G027282, were found in these two miR-eQTL regions individually on chromosome 5.

**Table 2:** Most significant associated SNPs of miRNA GWAS results

Associated miRNA	Chr^a^	Position	Marker	*r*^2^	*p*-value
miR156k-5p	6	96,303,448	6_96,303,448	0.25	2.06E-13
miR159a-3p	6	111,739,862	6_111,739,862	0.18	8.13–10
miR390a-5p	5	2,799,777	5_2799777	0.19	2.08E-10
miR396e-5p	5	214,388,579	5_214388579	0.24	6.44E-13

^a^ chromosome

**Table 3:** Genes within the strong associated SNPs region

miRNA	SNP	*p*-value	Chr^a^	Gene ID	Maize annotation	Arabidopsis hit	Arabidopsis orthology	Rice hit	Rice orthology
miR156k-5p	S6_95875558	3.45E-09	6	GRMZM2G049730	hagtf27-GNAT-transcription factor 27	AT4G37580.1	NAT superfamily protein	LOC_Os06g44100.1	HLS putative
	S6_96005268	4.01E-10	6	GRMZM2G014119	ubi3-ubiquitin3	AT1G31340.1	related to ubiquitin 1	LOC_Os09g25320.1	ubiquitin family protein
	S6_96007934	1.25E-07	6	GRMZM2G326734	NON	NON		LOC_Os06g44060.1	phospholipase D. Active site motif family
	S6_96096148	6.49E-11	6	GRMZM2G171317	dmag1 - DNA-3-methyladenine glycosylase1	AT5G57970.1	DNA glycosylase superfamily protein	LOC_Os06g44050.1	methyladenine glycosylase
	S6_96103728	2.93E-11	6	GRMZM2G171328	NON	AG5G54830.1	DOMON domain / dopamine beta-monooxygenase N-terminal	LOC_Os06g44040.1	DOMON domain
	S6_96289445	2.81E-10	6	GRMZM5G806818	NON	NON	NON	NON	NON
	S6_96349269	3.03E-11	6	AC233936.1_FG003	NON	AT5G25150.1	(TAF5) TBP-associated factor 5	LOC_Os06g44030.2	WD domain G-beta repeat domain
	S6_96352871	4.94E-11	6	AC208711.3_FG005	NON	NON		LOC_Os08g09890.1	Expressed protein
miR390a-5p	S5_2799777	2.083259e-10	5	GRMZM2G108694	NON	AT2G47430.1	(CKI1) Signal transduction histidine kinase	LOC_Os06g08450.1	histidine kinase putative expressed
miR396e-5p	S5_214385721	1.69E-10	5	GRMZM2G027282	NON	AT3G05530.1	(ATS6A.2, RPT5A) regulatory particle triple-A ATPase 5A	LOC_Os06g07630.1	26S protease regulatory subunit 6A

^a^ chromosome

### The effect of miR-eQTLs on their target genes

It is evident that miRNAs control plant development by regulating their target genes. To understand whether miR-eQTLs identified in this study could affect the expression of the associated miRNAs and their target genes, the expression levels of miRNA and their target genes in the maize panel were obtained from the same mature leaf RNA samples for both miRNA library and mRNA library construction. In this way, we could ensure the accuracy of the expression level of miRNA relative to their target genes. To understand the regulation of miRNAs on their target genes, the expression levels of miRNAs and their target genes were collected, separated and compared based on the genotypes of significantly associated SNPs in the identified miR-eQTLs individually (Table 4). For miR-eQTL to contribute to miR156k-5p, the miRNA expression level was lower when the most associated SNP type (marker 6_96,303,448) was A but higher when the SNP type was G (Fig. 3c). In contrast, the target gene *sbp29* of miR156k-5p displayed a higher expression level if the SNP type was A and a lower level when the SNP type was G (Fig. 3d). Therefore, a significantly negative relationship between the expression levels of miR156k-5p and the target gene sbp29 was identified. This indicated that miRNA might exert its control over target genes by suppression. A similar trend was shown between miR390a-5p (Fig. 5c) and its target gene GRMZM5G806469 (Fig. 5d). On the other hand, the expression levels of miR159a-3p and its target gene GRMZM2G416652 (MYB-transcription factor 122, *myb122*) were both lower when the strongest associated SNP type was A and higher when the SNP type was C (Fig. 4b and c), indicating a positive relationship between them. The same positive relationship was also discovered between miR396e-5p (Fig. 6c) and its target genes GRMZM2G478709, GRMZM2G124566 (GRF-transcription factor 9, *grftf9*), and GRMZM2G033612 (GRF-transcription factor 5, *grf5*) (Fig. 6d to f). In summary, the miRNAs associated with miR-eQTLs identified in this study might exert their control over their targets through mechanisms of both suppression and enhancement.

**Table 4:** Expression trend between miRNA and its target genes

Associated miRNA	SNP type	miRNA expression level	*p*-value	Target gene	Target gene expression level	*p*-value	Target region	Trend
miR156k-5p	A	low	7.1e-09	sbp29	high	0.018	3’UTR	Negative
	G	high			low			
miR390a-5p	G	high	6.3e-0.7	GRMZM5G806469	low	0.088	3’UTR	Negative
	T	low			high			
miR159a-3p	A	low	1.1e-0.5	myb122	low	0.05	CDS	Positive
	C	high			high			
miR396e-5p	C	high	0.00046	GRMZM2G478709	high	0.026	CDS	Positive
	T	low			low			
				grftf9	high	0.024		
					low			
				grf5	high	0.01		
					low			

## Discussion

### Highly expressed miRNAs and coexpressed miRNAs

This study aimed to identify miR-eQTLs in a specific developmental stage using the mature leaf tissue (leaf at the flowering time) of the maize panel as RNA sources for both mRNA and small RNA libraries. Gene regulation at this stage is not only important for maize yield but also a very complex network with many genes controlled in a highly and precisely regulated way. The highly expressed miRNAs might be different among plant tissues. Zhang et al. [18] studied the miRNA expression levels of 26 maize miRNA families among five different tissues (root, seedling, tassel, ear and pollen). It was shown that the highest expressed miRNAs varied based on different maize tissues, suggesting tissue- and developmental stage-specific miRNA expression [18]. In this study, miRNAs of the mature leaf tissue analyzed showed that the expression level of miR159a-3p was the highest, while miR171d-3p was the lowest, which was quite different from the reported miRNA types in a previous study [18]. This might be caused by the difference in environmental factors in the maize field including water, sunlight, winds, and soil conditions between materials reported in Zhang et al. [18] and this study. According to the pervious study, the correlation between different miRNAs which physically clustered miRNAs (< 10 kb) showed a higher positive correlation in humans. It was suggested that coexpression might occur among those miRNAs in the proximity of the genomic regions [39]. To understand if 31 highly expressed miRNAs were response to the environment or the physically clustered, the coexpression of highly expressed miRNA were detected. In our study, none of those miRNAs with a strong positive correlation were located on the same chromosome, even though some of them were within the same miRNA family. This indicated that the physical locations of miRNAs might not be a major factor responsible for the coexpression of miRNAs during the maize mature leaf developmental stage. With regard to the coexpression of miRNAs, the function of the miRNA target gene is one of the possible reasons why miRNAs would be coexpressed within the same miRNA family or between members of different miRNA families. For example, coexpression of members within or between miR166 and miR167 was identified in this study, and maize miR166 and miR167 families have been reported to all target bZIP transcription factors and auxin response factors, respectively [40]. The bZIP transcription factors have been reported to be involved in pathogen defense responses, light and stress signaling, seed maturation, and flower development [40]. Auxin response factors were reported to affect leaf and flower development [41]. During maize plant growth, leaves might need bZIP transcription factors to respond to stress and auxin response factors for developmental regulation. This might explain why the coexpression within the same family members of miR166 (miR166a-3p vs miR166e, miR166e vs miR166k-3p, miR166a-3p vs miR166I-3p, miR166k-3p vs miR166I-3p) and miR167 (miR167a-5p vs miR167e-5p) and members between different miRNAs (miR166k-3p and miR167h-3p) were detected in this study. On the other hand, Sunkar [11] reported that maize miR166 and miR167 showed similar expression patterns after treatment with hypoxia stress, which might also explain the coexpression of members between the two miRNA families. Nevertheless, the target gene functions of miR398, miR408, and miR528, which were coexpressed in this study, remain unknown, and more experiments are required to understand the relationship between members of these miRNA families. Furthermore, the maize panel we used could be distinguished into six subgroups based on their genetic background [42]. We have tested if the six genetic groups affect the miRNA expression of the final four targeted miRNAs, and found that no differences was shown among six different maize subgroups.

### The trans miR-eQTLs and their candidate genes

Trans-regulators tend to be identified more than cis-regulators when they contribute to miRNA expression in fibroblasts [33] and whole blood [37] in humans. Similarly, 70% of found eQTLs contributed as trans-regulators in maize, rice, and brassica rape (reviewed by [32]). Furthermore, the trans-eQTLs for regulatory genes were mostly found > 100 kb away (71.6%) from their regulated target genes, and 8% were located on different chromosomes in maize [43]. It has also been discovered that trans-eQTLs are highly specific to the environment in *C. elegans* [44] and plants [32]. In this study, the identified eQTLs were trans-regulators, with one located 100 kb away and three located on different chromosomes to their target miRNA. This suggests that each eQTL detected in this study might be specific to a certain environment. The four miRNAs identified with eQTLs in the present study indicate that miRNAs play an important role in plant development and environmental adaptation. For instance, miR156k and miR159a have been proven to respond to salt stress in maize roots [45] or change their expression under hormone depletion and light exposure in maize somatic embryogenesis [46]. In addition, miR396e has been revealed to respond to UV-B regulation in maize leaves [47], and the miR390 family has been found to accumulate during the development of the maize shoot apex [48]. Maize growth in the field may owe its endurance to the above special environmental factors. This could explain why the eQTLs we found were all trans-regulators. Furthermore, three targeted miRNAs in our study, miR156, miR159 and miR390 have been found functional involved in flowering time, a sensitive trait that response to the environment, in which played important roles to regulate the transcription factors and control the time of transition from the vegetative stage into the productive stage in a previous report by Spanudakis and Jackson [49]. This may indirectly provide the evidence that trans-regulators identified in this study have shown the interaction with the environment. However, more experiments were required in the future to validate the assumptions.

A total of eight candidate genes were found within the genomic region of miR-eQTL that contribute to the expression variance of miR156k-5p (Table 3). One of the candidate genes, *hagtf27* (GRMZM2G049730), showed homology to histone acetyltransferases involved in several metabolic pathways in plants [50], such as lipid metabolism and jasmonic acid biogenesis in cotton [51]. The function of ubiquitin family proteins is a key characteristic of cell autophagy and proteasome metabolism [52]. Another candidate gene, dmag1 (GRMZM2G171317), is a DNA glycosylase superfamily protein participating in the DNA damage repair pathway [53]. Additionally, the candidate gene GRMZM2G171328 showed homology to a DOMON domain-containing protein in rice, indicating an important role in heme and sugar recognition in maize cells [54]. The candidate gene AC233936.1_GF003, similar to proteins with the WD domain, has been reported to have multiple functions in genome integrity and cell cycle progression [55]. It has been reported that DNA damage could regulate miRNA expression through a p53-dependent pathway or modulation of the steps of miRNA processing and maturation. MiRNAs were also shown to be related to DNA damage repair and apoptosis [53]. The sampled tissue in this study was mature leaves in which cells might undergo DNA damage, even the progression of autophagy and apoptosis. This might explain why these candidate genes were identified within the miR-eQTL genomic region and contribute to the expression of miR156k-5p in this study. For another two miR-eQTLs, MiR390a-5p and miR396e-5p, no annotation of the candidates GRMZM2G108694 and GRMZM2G027282 in maize genome databases could be found. However, the translated sequences of GRMZM2G108694 showed homology to Arabidopsis cytokinin-independent 1 (CKI 1), which functions as a cytokinin receptor [56]. In addition, the translated sequences of GRMZM2G027282 showed homology with Arabidopsis ATS6A.2 and RPT5A, which played an essential role in gametophyte development [57]. The expression of these genes has no significant correlation to the miRNA expression after analysis by Pearson’s correlation coefficient. This suggested that there might be other factors affecting miRNA expression in additon to the genes within the eQTL region.

### The possible regulatory mechanisms of the four miR-eQTLs and their target genes

The relationship between miRNAs and their target genes is of great interest. This study identified that miR-eQTLs contribute to miRNAs, which showed both negative (miR156k-5p, miR390a-5p) and positive (miR159a-3p, miR396e-5p) ways of regulating their target genes individually (Table 4, Fig. S2). In humans, the localization of miRNA has been reported to play a key role in determining the mechanisms of target gene regulation [58]. In this study, the target binding sites of miR156k-5p and miR390a-5p were all at the 3’UTR of miRNA target genes. On the other hand, miR159a-3p and miR396e-5p were at the CDS of miRNA target genes. For the negative regulation of miRNAs on their target genes, such as miR156k-5p and miR390a-5p in this study, it has been reported that human miRNAs repress their target genes through RNA degradation and translational repression pathways. For positive regulation, in humans, miRNA was reported to accumulate in the cell nucleus [59]. Several types of human miRNAs in which target gene expression is upregulated have been reported, including promoter-targeting, TATA-box-activating and enhancer-associated miRNAs. However, this study identified positive regulation of miR159a-3p and miR396e-5p through binding the CDS of target genes that did not include any type of upregulated miRNA described above [60]. This indicated that the positive regulation mechanisms of miRNAs in maize might be different from those in humans, and this might be a novel or plant-specific regulation method.

## Conclusion

miRNAs have been proven to universally exist in animals and plants. Its biogeneration methods, functions, and target genes have also been investigated carefully. In this study, we estimated the correlation among 31 highly expressed miRNAs using NGS on small RNA libraries derived from mature leaf samples of the maize panel (200 maize lines) to understand the network of miRNAs. A strong positive correlation was found within and between miRNA families, and environmental factors might be the cause of the coexpression. Four miR-eQTLs contributing to miR156k-5p, miR159a-3p, miR390a-5p and miR396e-5p were identified by GWAS analysis on the maize panel. This study is the first report on the identification of maize miR-eQTLs and demonstrates that both negative and positive regulatory relationships exist between miR-eQTLs and their target genes. The positive regulation of miR159a-3p and miR396e-5p on the targets suggests that some plant miRNAs might regulate their target genes positively by a mechanism different from that of humans.

## Methods

### Plant materials and total RNA extraction

The maize 282 panel as mentioned in the previous study [61] was applied. The mature leaf tissues (half individuals of each row flowering in the field) of each maize line was collected with two different date (8 August 2014 and 26 August 2014) to minimize the effects from the collection date. The leaf defined as matured when at least half of individuals per genotype were flowering in the field before the two harvest dates above. The second leaf from tassel was collected and the leaf section was made based on 1 cm square in the center of the leaf, three plants were combined per genotype for further analysis. The total RNA was extracted in a previous report [61]. Total RNA was prepared for further small RNA libraries.

### Small RNA library development and Next Generation Sequencing

For developing small RNA libraries through the NEB small RNA library prep kit, a 2 ng fraction of the total RNA of each sample from 282 maize lines was prepared and shipped to the company “Global Biologics”. Small RNA libraries were diluted to 4.3 mM individually, and 96 libraries were pooled together for next-generation sequencing by NextSeq 500 with the condition of 75 bp, single end. The raw reads were uploaded to NCBI (BioProject ID: PRJNA599406).

### Library analysis and estimation of miRNA expression

A total of 200 out of the original 282 libraries derived from mature leaf samples were selected based on the quality of sequencing, and reads with lengths between 20 and 32 bp were kept after trimming the adapter. Then, reads were eliminated from rRNA, tRNA, and snoRNA by the Bowtie alignment tool [62]. From miRbase (http://www.mirbase.org/), 203 maize unique mature miRNA sequences were downloaded as a reference of mature miRNAs. Reads from libraries were classified by length and matched with the relative length of the miRNA reference. For example, reads lengths equal to 20 bp were separated from each library and matched with an miRNA reference of length equal to 20 bp. Highly expressed miRNAs were selected and defined as the sum of matched read numbers among all maize lines in the panel higher than 2000 and kept for further analysis. Data transformation was performed by Boxcox [63] with the matched read number as a phenotype of the specific miRNA. The correlation among all highly expression miRNA was performed by the Pearson correlation coefficient under R program.

### Multidimensional scaling (MDS) and Genome-wide association study (GWAS)

MDS was performed by the R program to identify the distance relationship among all samples based on the highly expressed miRNAs. Maize hapmap 3.21 was chosen as a genotype [64] and imputed by the K-nearest neighbor imputation (KNNi) method [65]. GWAS analysis was performed by TASSEL 5.0 [66] under GLM with 5 genetic PCAs plus 2 latent PCAs as covariates to control the population structure. SNPs were kept at only a *p*-value lower than 0.1 after the analysis. To test if the miRNA and target gene show the different expression level based on the highest associated SNP, T-test was performed under R program.

## Supplementary information

**Additional file 1: Fig. S1.** Correlation of 31 highly expressed miRNAs among 200 maize lines.

**Additional file 2: Fig. S2.** Regulation network among most significant SNP, miRNA and miRNA target genes. a. Regulation network of miR156k-5p and its target genes based on the most significant SNP. b. Regulation network of miR159a-3p and its target genes based on the most significant SNP. c. Regulation network of miR390a-5p and its target genes based on the most significant SNP. d. Regulation network of miR396e-5p and its target genes based on the most significant SNP.

## Data Availability

Not applicable.

## Abbreviations

AGO1: Argonaute1
DCL-1: Dicer like-1
DCL-4: Dicer like-4
eQTL: expressed quantitative traits loci
GLM: general linear model
GWAS: genome-wide association study
HEN1: Hua-Enhancer1
HST: HASTY
IP: Immunoprecipitation
KNNi: K-Nearest Neighbor imputation
miRNA: microRNA
miR-eQTL: miRNA expression-related quantitative traits loci
RdDM: RNA dependent DNA methylation
RISC: RNA-induced ribonucleoprotein silencing complex
UTR: untranslated region

## References

[1] Rogers K, Chen X (2013). Biogenesis, turnover, and mode of action of plant microRNAs. Plant Cell.

[2] Unver T, Namuth-Covert DM, Budak H. Review of current methodological approaches for characterizing microRNAs in plants. Int J Plant Genomics. 2009;2009. 10.1155/2009/262463 PMC2760397 19834623

[3] Ha M, Kim VN (2014). Regulation of microRNA biogenesis. Nat Rev Mol Cell Biol.

[4] Jones-Rhoades MW, Bartel DP (2004). Computational identification of plant microRNAs and their targets, including a stress-induced miRNA. Mol Cell.

[5] Ambros V (2004). The functions of animal microRNAs. Nature.

[6] Wilbert ML, Yeo GW (2011). Genome-wide approaches in the study of microRNA biology. Wiley Interdiscip Rev Syst Biol Med.

[7] Jones-Rhoades MW, Bartel DP, Bartel B (2006). MicroRNAs and their regulatory roles in plants. Annu Rev Plant Biol.

[8] Bartel D, Lewis B, Jones-Rhoades M, Burge C: Systems and methods for identifying miRNA targets and for altering miRNA and target expression. In: Google Patents; 2006.

[9] An W, Gong W, He S, Pan Z, Sun J, Du X (2015). MicroRNA and mRNA expression profiling analysis revealed the regulation of plant height in Gossypium hirsutum. BMC Genomics.

[10] Fujii H, Chiou T-J, Lin S-I, Aung K, Zhu J-K (2005). A miRNA involved in phosphate-starvation response in Arabidopsis. Curr Biol.

[11] Sunkar R, Li Y-F, Jagadeeswaran G (2012). Functions of microRNAs in plant stress responses. Trends Plant Sci.

[12] Chen X (2004). A microRNA as a translational repressor of APETALA2 in Arabidopsis flower development. Science.

[13] Ding D, Wang Y, Han M, Fu Z, Li W, Liu Z, Hu Y, Tang J (2012). MicroRNA transcriptomic analysis of heterosis during maize seed germination. PLoS One.

[14] Kang M, Zhao Q, Zhu D, Yu J (2012). Characterization of microRNAs expression during maize seed development. BMC Genomics.

[15] Jin X, Fu Z, Lv P, Peng Q, Ding D, Li W, Tang J (2015). Identification and characterization of microRNAs during maize grain filling. PLoS One.

[16] Barber WT, Zhang W, Win H, Varala KK, Dorweiler JE, Hudson ME, Moose SP (2012). Repeat associated small RNAs vary among parents and following hybridization in maize. Proc Natl Acad Sci.

[17] Ding H, Gao J, Luo M, Peng H, Lin H, Yuan G, Shen Y, Zhao M, Pan G, Zhang Z (2013). Identification and functional analysis of miRNAs in developing kernels of a viviparous mutant in maize. Crop J.

[18] Zhang L, Chia J-M, Kumari S, Stein JC, Liu Z, Narechania A, Maher CA, Guill K, McMullen MD, Ware D (2009). A genome-wide characterization of microRNA genes in maize. PLoS Genet.

[19] Zhai L, Liu Z, Zou X, Jiang Y, Qiu F, Zheng Y, Zhang Z (2013). Genome-wide identification and analysis of microRNA responding to long-term waterlogging in crown roots of maize seedlings. Physiol Plant.

[20] Zhang Z, Lin H, Shen Y, Gao J, Xiang K, Liu L, Ding H, Yuan G, Lan H, Zhou S (2012). Cloning and characterization of miRNAs from maize seedling roots under low phosphorus stress. Mol Biol Rep.

[21] Trevisan S, Nonis A, Begheldo M, Manoli A, Palme K, Caporale G, Ruperti B, Quaggiotti S (2012). Expression and tissue-specific localization of nitrate-responsive miRNAs in roots of maize seedlings. Plant Cell Environ.

[22] Xu Z, Zhong S, Li X, Li W, Rothstein SJ, Zhang S, Bi Y, Xie C (2011). Genome-wide identification of microRNAs in response to low nitrate availability in maize leaves and roots. PLoS One.

[23] Wu F, Shu J, Jin W (2014). Identification and validation of miRNAs associated with the resistance of maize (*Zea mays* L.) to Exserohilum turcicum. PloS one.

[24] Thiebaut F, Rojas CA, Grativol C, Motta MR, Vieira T, Regulski M, Martienssen RA, Farinelli L, Hemerly AS, Ferreira PC (2014). Genome-wide identification of microRNA and siRNA responsive to endophytic beneficial diazotrophic bacteria in maize. BMC Genomics.

[25] Mitchell-Olds T (2010). Complex-trait analysis in plants. Genome Biol.

[26] Korte A, Farlow A (2013). The advantages and limitations of trait analysis with GWAS: a review. Plant Methods.

[27] Ogura T, Busch W (2015). From phenotypes to causal sequences: using genome wide association studies to dissect the sequence basis for variation of plant development. Curr Opin Plant Biol.

[28] Morris GP, Ramu P, Deshpande SP, Hash CT, Shah T, Upadhyaya HD, Riera-Lizarazu O, Brown PJ, Acharya CB, Mitchell SE (2013). Population genomic and genome-wide association studies of agroclimatic traits in sorghum. Proc Natl Acad Sci.

[29] Dhanapal AP, Ray JD, Singh SK, Hoyos-Villegas V, Smith JR, Purcell LC, King CA, Cregan PB, Song Q, Fritschi FB (2015). Erratum to: genome-wide association study (GWAS) of carbon isotope ratio (δ 13 C) in diverse soybean [Glycine max (L.) Merr.] genotypes. Theor Appl Genet.

[30] Wang M, Wang Q, Zhang B (2013). Response of miRNAs and their targets to salt and drought stresses in cotton (Gossypium hirsutum L.). Gene.

[31] Cubillos FA, Coustham V, Loudet O (2012). Lessons from eQTL mapping studies: non-coding regions and their role behind natural phenotypic variation in plants. Curr Opin Plant Biol.

[32] Holloway B, Luck S, Beatty M, Rafalski J-A, Li B (2011). Genome-wide expression quantitative trait loci (eQTL) analysis in maize. BMC Genomics.

[33] Borel C, Deutsch S, Letourneau A, Migliavacca E, Montgomery SB, Dimas AS, Vejnar CE, Attar H, Gagnebin M, Gehrig C (2011). Identification of cis-and trans-regulatory variation modulating microRNA expression levels in human fibroblasts. Genome Res.

[34] Dong H, Luo L, Hong S, Siu H, Xiao Y, Jin L, Chen R, Xiong M (2010). Integrated analysis of mutations, miRNA and mRNA expression in glioblastoma. BMC Syst Biol.

[35] Civelek M, Hagopian R, Pan C, Che N, Yang W-P, Kayne PS, Saleem NK, Cederberg H, Kuusisto J, Gargalovic PS (2013). Genetic regulation of human adipose microRNA expression and its consequences for metabolic traits. Hum Mol Genet.

[36] Siddle KJ, Deschamps M, Tailleux L, Nédélec Y, Pothlichet J, Lugo-Villarino G, Libri V, Gicquel B, Neyrolles O, Laval G (2014). A genomic portrait of the genetic architecture and regulatory impact of microRNA expression in response to infection. Genome Res.

[37] Huan T, Rong J, Liu C, Zhang X, Tanriverdi K, Joehanes R, Chen BH, Murabito JM, Yao C, Courchesne P (2015). Genome-wide identification of microRNA expression quantitative trait loci. Nat Commun.

[38] Liu H, Qin C, Chen Z, Zuo T, Yang X, Zhou H, Xu M, Cao S, Shen Y, Lin H (2014). Identification of miRNAs and their target genes in developing maize ears by combined small RNA and degradome sequencing. BMC Genomics.

[39] Chaulk SG, Ebhardt HA, Fahlman RP (2016). Correlations of microRNA: microRNA expression patterns reveal insights into microRNA clusters and global microRNA expression patterns. Mol BioSyst.

[40] Jakoby M, Weisshaar B, Dröge-Laser W, Vicente-Carbajosa J, Tiedemann J, Kroj T, Parcy F (2002). bZIP transcription factors in Arabidopsis. Trends Plant Sci.

[41] Li S-B, Xie Z-Z, Hu C-G, Zhang J-Z (2016). A review of auxin response factors (ARFs) in plants. Front Plant Sci.

[42] Flint-Garcia SA, Thuillet AC, Yu J, Pressoir G, Romero SM, Mitchell SE, Doebley J, Kresovich S, Goodman MM, Buckler ES (2005). Maize association population: a high-resolution platform for quantitative trait locus dissection. Plant J.

[43] Liu H, Luo X, Niu L, Xiao Y, Chen L, Liu J, Wang X, Jin M, Li W, Zhang Q (2017). Distant eQTLs and non-coding sequences play critical roles in regulating gene expression and quantitative trait variation in maize. Mol Plant.

[44] Snoek BL, Sterken MG, Bevers RP, Volkers RJ, van’t Hof A, Brenchley R, Riksen JA, Cossins A, Kammenga JE: Contribution of trans regulatory eQTL to cryptic genetic variation in *C. elegans*. BMC genomics 2017, 18(1):500. 10.1186/s12864-017-3899-8 PMC5492678 28662696

[45] Ding D, Zhang L, Wang H, Liu Z, Zhang Z, Zheng Y (2009). Differential expression of miRNAs in response to salt stress in maize roots. Ann Bot.

[46] Chávez-Hernández EC, Alejandri-Ramírez ND, Juárez-González VT, Dinkova TD (2015). Maize miRNA and target regulation in response to hormone depletion and light exposure during somatic embryogenesis. Front Plant Sci.

[47] Casati P (2013). Analysis of UV-B regulated miRNAs and their targets in maize leaves. Plant Signal Behav.

[48] Nogueira FT, Chitwood DH, Madi S, Ohtsu K, Schnable PS, Scanlon MJ, Timmermans MC (2009). Regulation of small RNA accumulation in the maize shoot apex. PLoS Genet.

[49] Spanudakis and Jackson: The role of microRNAs in the control of flowering time. J. Exper. Botany 2014, 65(2): 365–380. 10.1093/jxb/ert453 24474808

[50] Vetting MW, de Carvalho LPS, Yu M, Hegde SS, Magnet S, Roderick SL, Blanchard JS (2005). Structure and functions of the GNAT superfamily of acetyltransferases. Arch Biochem Biophys.

[51] Fu W, Shen Y, Hao J, Wu J, Ke L, Wu C, Huang K, Luo B, Xu M, Cheng X. Acyl-CoA N-acyltransferase influences fertility by regulating lipid metabolism and jasmonic acid biogenesis in cotton. Sci Rep. 2015;5. 10.1038/srep11790 PMC4488762 26134787

[52] Martens S, Bachmair A (2015). How cells coordinate waste removal through their major proteolytic pathways. Nat Cell Biol.

[53] Hu H, Gatti RA: MicroRNAs: new players in the DNA damage response. J Molecular Cell Biol 2010:mjq042. 10.1093/jmcb/mjq042 PMC3104011 21183529

[54] Iyer LM, Anantharaman V, Aravind L (2007). The DOMON domains are involved in heme and sugar recognition. Bioinformatics.

[55] Zhang C, Zhang F (2015). The multifunctions of WD40 proteins in genome integrity and cell cycle progression. J Genomics.

[56] Kakimoto T. CKI1, a histidine kinase homolog implicated in cytokinin signal transduction. Science. 1996:982–5. 10.1126/science.274.5289.982 8875940

[57] Gallois J-L, Guyon-Debast A, Lécureuil A, Vezon D, Carpentier V, Bonhomme S, Guerche P (2009). The Arabidopsis proteasome RPT5 subunits are essential for gametophyte development and show accession-dependent redundancy. Plant Cell.

[58] Zou Q, Liang Y, Luo H, Yu W: miRNA-mediated RNAa by targeting enhancers. In: RNA Activation. Springer; 2017: 113–125. 10.1007/978-981-10-4310-9_8 28639195

[59] Xiao M, Li J, Li W, Wang Y, Wu F, Xi Y, Zhang L, Ding C, Luo H, Li Y (2017). MicroRNAs activate gene transcription epigenetically as an enhancer trigger. RNA Biol.

[60] Huang V: Endogenous miRNAa: miRNA-mediated gene Upregulation. In: RNA Activation. Springer; 2017: 65–79. 10.1007/978-981-10-4310-9_5 28639192

[61] Kremling KA, Chen S-Y, Su M-H, Lepak NK, Romay MC, Swarts KL, Lu F, Lorant A, Bradbury PJ, Buckler ES (2018). Dysregulation of expression correlates with rare-allele burden and fitness loss in maize. Nature.

[62] Langmead B, Trapnell C, Pop M, Salzberg SL (2009). Ultrafast and memory-efficient alignment of short DNA sequences to the human genome. Genome Biol.

[63] Box GE, Cox DR. An analysis of transformations. J R Stat Soc Ser B Methodol. 1964:211–52.

[64] Bukowski R, Guo X, Lu Y, Zou C, He B, Rong Z, Wang B, Xu D, Yang B, Xie C: Construction of the third generation *Zea mays* haplotype map. GigaSci. 2018(7):1–12. 10.1093/gigascience/gix134 PMC5890452 29300887

[65] Money D, Gardner K, Migicovsky Z, Schwaninger H, Zhong G-Y, Myles S (2015). LinkImpute: fast and accurate genotype imputation for nonmodel organisms. G3: genes| genomes|. Genetics.

[66] Bradbury PJ, Zhang Z, Kroon DE, Casstevens TM, Ramdoss Y, Buckler ES (2007). TASSEL: software for association mapping of complex traits in diverse samples. Bioinformatics.

