# Supplementary figures and images for: Identification of miRNA-eQTLs in maize mature leaf by GWAS
Source: BMC Genomics. 2020 Oct 6;21:689. doi: 10.1186/s12864-020-07073-0 (PMC7541240; doi:10.1186/s12864-020-07073-0)

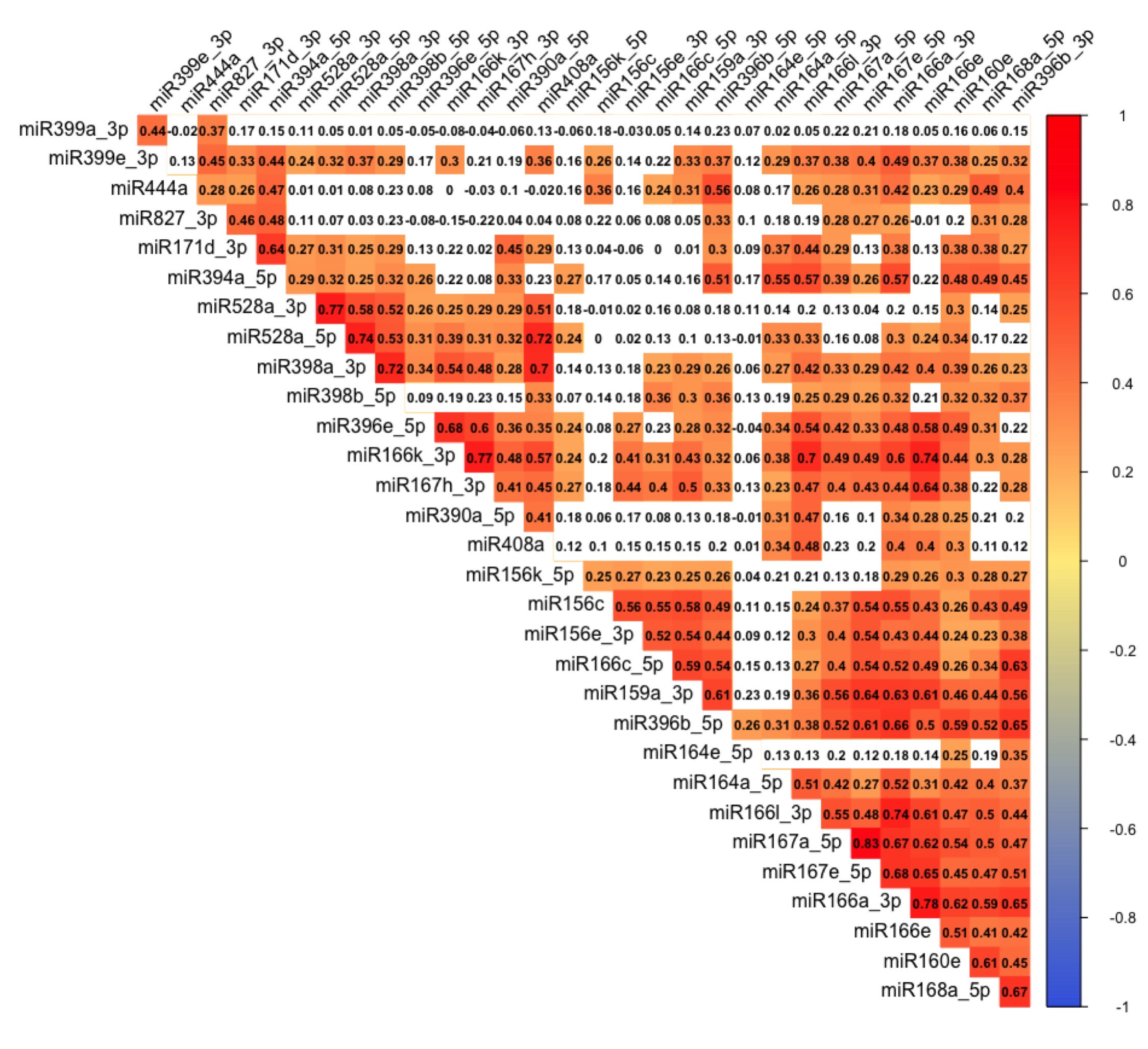

Supplement: Supplementary file 1 — Additional file 1: Fig. S1. Correlation of 31 highly expressed miRNAs among 200 maize lines. [file 12864_2020_7073_MOESM1_ESM.jpg]

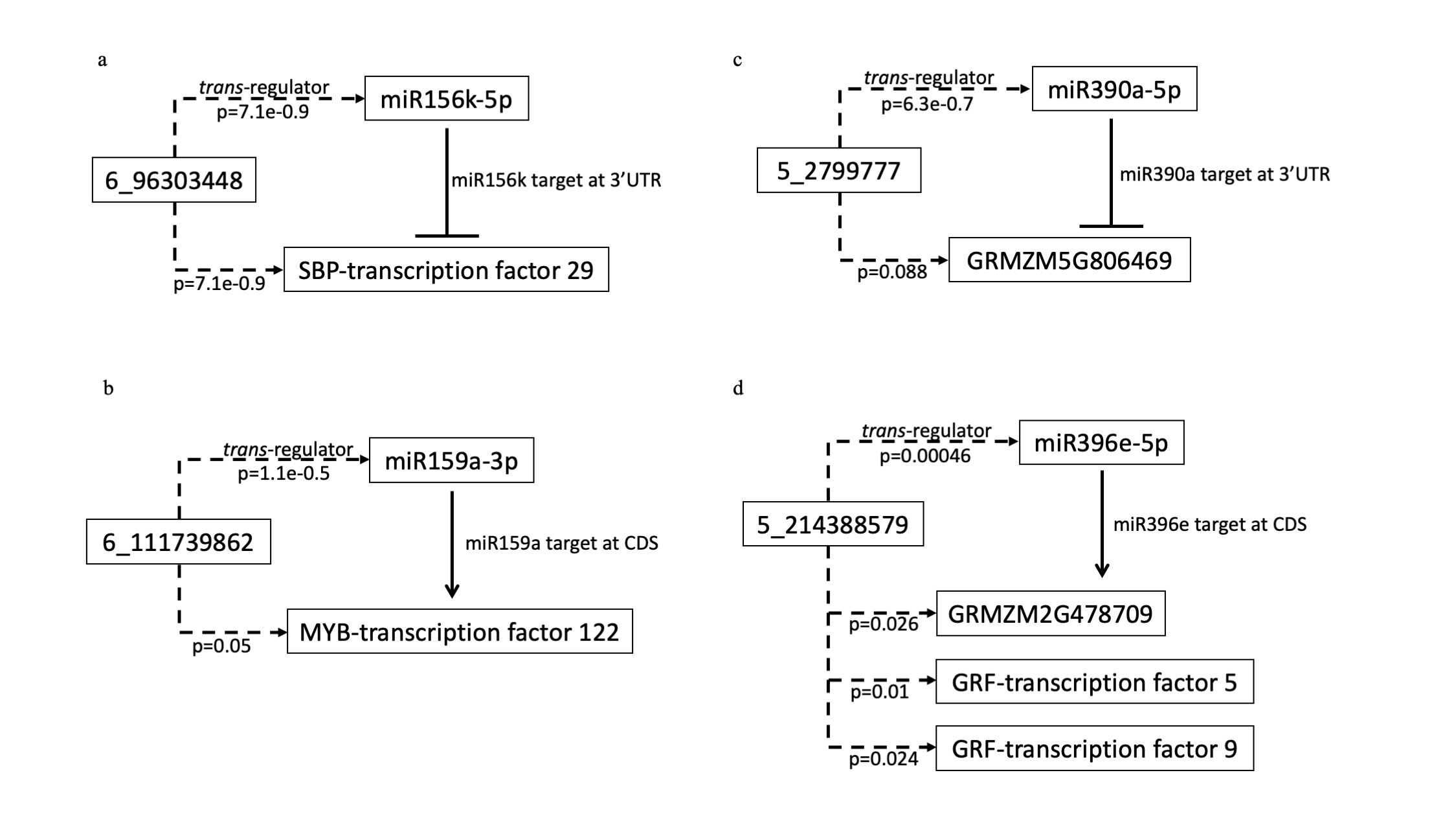

Supplement: Supplementary file 2 — Additional file 2: Fig. S2. Regulation network among most significant SNP, miRNA and miRNA target genes. a. Regulation network of miR156k-5p and its target genes based on the most significant SNP. b. Regulation network of miR159a-3p and its target genes based on the most significant SNP. c. Regulation network of miR390a-5p and its target genes based on the most significant SNP. d. Regulation network of miR396e-5p and its target genes based on the most significant SNP. [file 12864_2020_7073_MOESM2_ESM.jpg]
